# Factors Affecting Nonresponse Among Female Participants in the Korea Nurses’ Health Study: Longitudinal Cohort Survey Study

**DOI:** 10.2196/68038

**Published:** 2025-10-20

**Authors:** Young Taek Kim, Chiyoung Cha, Gumhee Baek, Bohye Kim, Bo Mi Song, Joong-Yeon Lim, Hyun-Young Park, Juh Hyun Shin

**Affiliations:** 1 Division for Gender-based Violence Research Korean Women's Development Institute Seoul Republic of Korea; 2 College of Nursing Ewha Research Institute of Nursing Science Ewha Womans University Seoul Republic of Korea; 3 Nursing Workforce Support Center Korean Nursing Association Seoul Republic of Korea; 4 Division of Population Health Research, Department of Precision Medicine National Institute of Health Cheongju Republic of Korea; 5 National Institute of Health Cheongju Republic of Korea; 6 School of Nursing George Washington University Washington, DC United States

**Keywords:** cohort studies, female, follow-up studies, logistic models, longitudinal studies, nonresponse, nurses

## Abstract

**Background:**

The major drawback of a cohort study design is the loss to follow-up, which increases selection bias and threatens external validity, particularly in online surveys. It is important to identify factors beyond population or demographics that influence nonresponse rates in cohort studies.

**Objective:**

This study aimed to examine the nonresponse rate and associated factors over a 10-year follow-up period among female participants in the Korea Nurses’ Health Study using data from the initial and subsequent surveys.

**Methods:**

The Korea Nurses’ Health Study recruited 20,613 female nurses in 2013 using simple random sampling. The participants were followed up 10 times through 2022. We identified the demographic, work-related, survey-related, and psychological characteristics of nonresponding nurses during the 10-year follow-up and compared them with those who continued to participate. Descriptive statistics, chi-square tests, and multivariate logistic regression models were used for the analysis.

**Results:**

The nonresponse rate of the follow-up surveys from the 2nd to the 11th survey varied between 25.5% (5258/20,613; second survey) and 61.2% (12,620/20,613; sixth survey). The influence of age, education, and the usability of survey websites on nonresponse lasted up to the 11th survey. Nurses in their 20s were less likely to respond to the follow-up surveys than those in their 30s. Those who had an associate degree and neutral feelings about the usability of the survey websites were less likely to respond to the follow-up surveys than those who were satisfied with the initial survey. The influence of geographical region, hospital size, and psychological factors—including stress, fatigue, and sleep disturbance—was evident from the second to the sixth survey.

**Conclusions:**

When designing and recruiting female nurse participants for community-based cohort studies, researchers should consider the factors that influence nonresponse and adopt tailored strategies based on demographic characteristics. In addition, improving the usability of survey websites is recommended to reduce nonresponses at follow-up in cohort studies involving female participants.

**International Registered Report Identifier (IRRID):**

RR2-DOI: https://doi.org/10.4178/epih.e2024048

## Introduction

### Background

A cohort study was conducted in 1935 in the United States to determine the incidence of tuberculosis [1]. Despite their costly and time-consuming nature, cohort studies have been recognized as valuable longitudinal research methods. Cohort studies have been widely conducted to identify associations between known and unknown risk factors for specific diseases [2]. One of the major drawbacks of the cohort study design is the loss of participants at follow-up. With awareness of the differences in health disparities and determinants of health between men and women, cohort studies have been conducted to identify salient factors related to health and illness among women across several countries [3-5]. In 2013, the Korea Nurses’ Health Study, a national cohort study of 20,613 female nurses aged between 20 and 45 years, was initiated to determine the sociodemographic, occupational, and lifestyle factors affecting the health and disease status of Korean women [6]. Follow-up surveys were conducted annually for more than 10 years, allowing the recognition of long-term patterns formed from early to middle adulthood. Participants accessed the Korea Nurses’ Health Study through an online platform, with invitations delivered via phone, SMS text messages, or email.

Nonresponse is a significant issue in longitudinal studies, with response rates varying widely across different populations. In general, a response rate of 50% is considered acceptable [7]. Studies of nurses in Western countries have often reported response rates below 30% [3,8-10]. Similarly, health surveys in the Netherlands reported nonresponse rates of 53% [11], while the Thai Nurse Cohort Study reported nonresponse rates of 39.8% [12]. In Korea, the Nurses’ Health Study, a prospective cohort study of women’s health that has been ongoing for more than 10 years, has a nonresponse rate of 50% [13]. In this study, we examined the nonresponse rate and its associated factors in up to 10 years of follow-up surveys.

### Literature Review

Nonresponse generally describes cases in which individuals do not participate in follow-up data collection [14] and the loss of participants over time, particularly in later data collection surveys, owing to factors such as loss of contact, incapacity, or refusal to continue participating [15]. Nonresponse can also occur when participants are lost to follow-up, choose to withdraw from the study, do not respond to follow-up surveys, or die during the study period [16]. This issue is particularly pronounced in online surveys, where a high rate of nonresponse increases the likelihood of nonrandom missing data [17]. Factors such as indifference to the study, rapidly changing health care environments, and illness might also contribute to nonresponse [3,17]. Previous cohort studies have examined factors related to nonresponse, with a focus on demographic factors, such as age [18], gender [19], education level [19-21], socioeconomic status [19], and health status [22]. However, participation in a survey can also be influenced by psychological factors, including the presence of stressful life events [23], depression, and anxiety disorders [21], along with participants’ perceptions of the survey, which have rarely been studied. This is particularly significant, as most previous cohort studies identifying factors associated with nonresponse have focused on individuals with health issues [21,24] or older adult populations [25,26]. Moreover, survey satisfaction and the time required to complete the surveys are factors that can influence future participation in cohort studies. Participants who were satisfied with the survey process were more likely to participate in a follow-up survey [23]. Conversely, long or burdensome surveys may increase nonresponse in subsequent surveys [8]. Thus, a comprehensive understanding of psychological and survey-related factors is crucial to address nonresponse rates in cohort studies.

## Methods

### Design and Participants

The Korea Nurses’ Health Study, initiated in 2013 with 20,613 participants, was originally developed based on the Nurses’ Health Study 3 conducted in the United States [10], which is the oldest cohort study on women’s health involving nurses as participants. For the Korea Nurses’ Health Study, registered nurses aged 20 to 45 years working at hospitals across Korea at the time of data collection were recruited through simple random sampling. According to the Korean Nursing Association, 157,569 women of childbearing age were identified as nurses. The target sample size was determined based on population parameters and was calculated to be 20,000 female nurses, considering a 95% CI with a 1% permissible error [6]. After recruiting 20,613 registered nurses in the first survey in 2013, follow-up surveys were subsequently conducted: second survey (March 2014 to April 2019), third survey (November 2014 to April 2019), fourth survey (September 2015 to April 2019), fifth survey (June 2016 to April 2019), sixth survey (September 2017 to April 2019), seventh survey (December 2018 to April 2019), eighth survey (October 2019 to April 2020), ninth survey (October 2020 to April 2021), 10th survey (October 2021 to August 2022), and 11th survey (March 2022 to April 2023).

Participants in the initial survey were invited to participate in the follow-up surveys. This approach ensured high response rates without affecting the consistency of response and nonresponse rate calculations because the timing of survey availability did not influence participant responses [13]. While deaths during the study period were not specifically investigated, the number of nurses eligible to respond changed over time due to refusals to continue follow-up contact. Participants who left the nursing field during the study period were included in follow-up surveys to capture changes in health outcomes and ensure that the study reflected the broader scope of women’s health. In addition, the participants were notified through SMS text messages and email reminders to encourage their participation. Surveys were accessible via both web and mobile platforms, ensuring convenience for participants.

### Measures

On the basis of the literature, we included demographic and work-related characteristics, psychological variables, and survey-related characteristics from the initial survey to examine the factors associated with nonresponse in the follow-up surveys. The participants self-reported these characteristics throughout the survey.

#### Demographic and Work-Related Characteristics

The demographic characteristics included age and geographical region, categorized as Seoul (the capital), other metropolitan cities, and smaller cities or rural areas. Work-related characteristics included hospital size (number of beds); job position (manager or head nurse and charge or staff nurse); and work unit (special unit, general ward, or delivery room).

#### Psychological Variables

Four scales were used to measure the psychological variables: depression, stress, fatigue, and sleep. Depressive symptoms were measured using a nine-item, 4-point Likert scale from the Patient Health Questionnaire [27]. Possible scores range from 0 to 27, with higher scores indicating more severe depressive symptoms. On the basis of a previous study, a cutoff score of 10 was used for no to mild and moderate to severe symptoms [27]. Its sensitivity and specificity were 88% [27]. In this study, the Cronbach α was 0.892. Stress was measured using the Perceived Stress Scale (PSS)-4 [28], which consists of four 5-point Likert-type items. The possible scores range from 0 to 16, with higher scores indicating higher stress levels. The cutoff scores were determined based on the literature, and the top tertile of the scores (≥11) was categorized as *high stress*, with the other tertiles considered as *low stress* [29]. The PSS-4 has been confirmed to be reliable and valid compared to the original 14-item PSS [28]. Cronbach α was 0.691. Fatigue was measured using the Chalder Fatigue Scale [30], which comprises 11 items rated on a 4-point Likert scale. The possible scores range from 0 to 33, with higher scores indicating higher levels of fatigue. Scores between 0 and 16 were categorized as low, those between 17 and 21 as moderate, and those above 22 as high fatigue [30]. In this study, Cronbach α was 0.918. Sleep disturbance was measured using the Jenkins Sleep Questionnaire [31], which evaluates sleep problems during the preceding 4 weeks. This scale comprises 4 items rated on a 6-point Likert-type scale. Scores range from 0 to 20, with higher scores indicating greater sleep disturbance. On the basis of the cutoff point of 12 or more, which indicates severe sleep problems, participants were classified into groups with little sleep disturbance and those with high frequency of sleep disturbance (<12 and ≥12) [31]. In this study, the Cronbach α was 0.875. The collinearity of the 4 psychological variables was tested using the Pearson correlation coefficient (*r*<0.4 for all 4 psychological variables).

#### Survey-Related Characteristics

Survey-related characteristics included 3 items: time spent on survey completion, usability of the survey website, and overall impression of the survey website. Time spent completing the survey was categorized as <15 minutes, 15 to 30 minutes, and ≥30 minutes. Usability and overall impressions of the survey website were assessed using end-of-survey questions, such as “What is your overall impression of this survey website?” and “What was your experience using the survey website?” The responses were categorized as unsatisfactory, neutral, or satisfactory.

### Data Analysis

Descriptive statistics were used to examine nonresponse rates and factors. Chi-square tests were used to compare the respondent and nonrespondent groups using initial survey data. A fully adjusted logistic regression model with available variables from the initial survey was used to identify factors associated with nonresponse. Missing data were excluded from analyses involving these variables. SPSS software (version 29; IBM Corp) was used for statistical analysis.

### Ethical Considerations

For the initial survey in 2013 and the follow-up surveys until 2015, the institutional review board of the National Institute of Health in Korea reviewed and approved the study (#2013-03CON-03-P, 2014-05EXP-01-1C-A, 2014-08EXP-01-2C-A, and 2015-05-EXP-04-3C-A). For the follow-up surveys conducted in 2016, the Ewha Womans University Institutional Review Board reviewed and approved the study (#117-4, EWURB-17-3.0-20170901, ewha-201904-0012-01, ewha-201904-0012-09, ewha-201904-0012-12, and ewha-201904-0012-15). The Korea Nurses’ Health Study survey instrument was meticulously designed by integrating questions from various validated sources to ensure comprehensive coverage of relevant topics. The quality of the survey was ensured through a rigorous pilot testing phase with nurses and expert groups to verify the clarity, relevance, and logical sequence of the questions. This process helped identify and rectify potential issues, such as redundancy and omission of important inquiries. No essential items from the original tools were omitted during the integration of questions from multiple sources. In cases where redundancy was identified, questions were asked only once to streamline the survey without compromising its psychometric properties. The questionnaire construction and refinement of the Korea Nurses’ Health Study have been described in greater detail elsewhere [6]. The participants provided informed electronic consent before participating in the baseline and follow-up surveys. The participants were informed of the research purpose, anonymity, and confidentiality. All data were fully deidentified before analysis, with access restricted to authorized investigators through secure servers in compliance with Korea Nurses’ Health Study data protection policies. Individual-level information was neither disclosed nor reported. Beverage vouchers were provided as an incentive.

## Results

### Overview

The nonresponse rates of the participants in the Korea Nurses’ Health Study are shown in Table 1. Participants from the baseline survey (N=20,613) were contacted for the follow-up surveys. The nonresponse rate varied between 25.5% (5258/20,613; second survey) and 61.2% (12,620/20,613; sixth survey), with the sixth survey having the highest nonresponse rate.

**Table 1:** Nonresponse rate of the Korea Nurses’ Health Study participants (first survey: N=20,613)

Survey number	Nonresponding participants, n (%)
2	5258 (25.5)
3	7765 (37.7)
4	9963 (48.3)
5	9086 (44.1)
6	12,620 (61.2)
7	11,955 (58.0)
8	10,360 (50.3)
9	9957 (48.3)
10	10,503 (51)
11	10,357 (47.9)

### Comparison of Respondent and Nonrespondent Groups

Differences between the respondent and nonrespondent groups from the second to the eleventh survey were compared based on participant characteristics from the first survey (Multimedia Appendix 1). In the initial survey, most participants were in their 20s (12,059/20,613, 58.5%) with a mean age of 29.4 (SD 5.9) years. All participants worked in hospitals, and 45.2% (9316/20,613) held a bachelor degree [13]. Age, education, stress, and perceived usability of the survey at baseline were found to be statistically different across the follow-up surveys, up to the 11th survey. Overall impressions of the survey website at baseline differed significantly in all follow-up surveys except the second. Nurses dissatisfied with the usability of the survey responded more to the second to fourth surveys than satisfied nurses, and nurses dissatisfied with the overall survey responded more to the third and fourth surveys. Geographical region and fatigue level in the baseline survey differed significantly between the respondent and nonrespondent groups up to the seventh survey. Hospital size at baseline was significantly associated with the response status in the fifth survey. The working units significantly differed until the third survey was conducted.

### Factors Affecting Nonresponse

A fully adjusted logistic regression analysis was performed to identify the factors associated with nonresponse in the follow-up surveys (Tables 2 and 3). Age, education level, and perceived usability of the survey website at baseline were significant factors that showed a consistent association with nonresponse throughout the 11th survey. In the third survey, nurses in their 20s were less likely to respond compared to those in their 30s (odds ratio [OR] 0.93, 95% CI 0.87-1.00); this association remained significant in all surveys through the 11th, except the second. In the second survey, nurses with an associate degree were less likely to respond than those with a bachelor degree (OR 0.84, 95% CI 0.79-0.91) or a master degree or higher (OR 0.75, 95% CI 0.64-0.88); this pattern remained significant through the 11th survey. Nurses who reported neutral feelings about the usability of the survey website were more likely not to respond in the second survey compared to those who perceived the survey website to be useful in the second survey (OR 0.90, 95% CI 0.81-0.99), and this association was consistently significant until the 11th survey.

The association between geographical region and hospital size lasted until the fifth and sixth surveys. In the second survey, nurses working in Seoul were more likely to respond compared to those working in metropolitan areas (OR 1.16, 95% CI 1.06-1.27) and other regions (OR 1.17, 95% CI 1.08-1.27). This association remained consistently significant until the sixth survey was conducted. Nurses who worked in hospitals with more than 300 beds were less likely to respond to the second survey compared to their counterparts (OR 1.40, 95% CI 1.29-1.52); this association was consistently significant until the fifth survey.

Age at baseline was a significant factor for nonresponse throughout the follow-up surveys, except for the second survey. Nurses in their 30s were less likely to be nonrespondents than those in their 20s in the third survey (OR 0.93, 95% CI 0.868-0.999), and this was consistently significant up to the 11th survey (OR 0.82, 95% CI 0.764-0.875).

**Table 2:** Fully adjusted logistic regression of second to sixth surveys for nonresponse in the Korea Nurses’ Health Study participants^a^.

Variables			Second survey, OR (95% CI)		Third survey, OR (95% CI)		Fourth survey, OR (95% CI)		Fifth survey, OR (95% CI)		Sixth survey, OR (95% CI)
**Age (y; reference: 20 to 29)**											
	30 to 39	1.02 (0.94-1.10)		0.93^b^ (0.87-1.00)		0.87^c^ (0.81-0.93)		0.83^d^ (0.78-0.90)		0.77^c^ (0.72-0.82)	
	≥40	0.90 (0.77-1.04)		0.92 (0.80-1.06)		1.13 (1.00-1.30)		1.00 (0.87-1.14)		0.87 (0.76-1.00)	
**Geographical region (reference: Seoul)**											
	Metropolitan cities	1.16^d^ (1.06-1.27)		1.24^c^ (1.15-1.34)		1.36^c^ (1.26-1.47)		1.26^c^ (1.17-1.36)		1.17^c^ (1.08-1.27)	
	Others	1.17^c^ (1.08-1.27)		1.22^c^ (1.13-1.31)		1.20^c^ (1.11-1.29)		1.17^c^ (1.09-1.26)		1.13^d^ (1.04-1.21)	
**Education (reference: associate degree)^c^**											
	Bachelor degree	0.84 (0.79-0.91)		0.85 (0.80-0.91)		0.84 (0.79-0.90)		0.82 (0.77-0.87)		0.79 (0.74-0.84)	
	Master degree or higher	0.75 (0.64-0.88)		0.72 (0.63-0.83)		0.76 (0.67-0.87)		0.75 (0.66-0.86)		0.77 (0.67-0.88)	
**Hospital size (reference: ≥300 beds)**											
	<300 beds	1.40^c^ (1.29-1.52)		1.36^c^ (1.26-1.46)		1.24^c^ (1.16-1.34)		1.15^c^ (1.07-1.24)		1.04 (0.96-1.12)	
**Job role (reference: charge or staff nurse)**											
	Manager or head nurse	1.04 (0.87-1.25)		0.99 (0.84-1.16)		1.09 (0.93-1.27)		1.05 (0.90-1.23)		1.13 (0.96-1.32)	
**Work unit type (reference: general ward)**											
	Special unit	1.01 (0.94-1.09)		0.97 (0.91-1.04)		0.98 (0.92-1.05)		1.03 (0.96-1.10)		1.03 (0.97-1.11)	
	Delivery room or others	1.11^b^ (1.01-1.23)		1.07 (0.98-1.17)		1.04 (0.95-1.13)		1.06 (0.97-1.15)		1.05 (0.96-1.14)	
**Depressive symptoms (reference: <10)**											
	≥10	1.00 (0.92-1.09)		0.98 (0.91-1.06)		0.98 (0.91-1.06)		0.94 (0.87-1.02)		0.91^b^ (0.84-0.99)	
**Stress (reference: <11)**											
	≥11	1.25^b^ (1.05-1.48)		1.16 (0.99-1.35)		1.12 (0.96-1.30)		1.17^b^ (1.00-1.37)		1.19^b^ (1.01-1.41)	
**Fatigue (reference: approximately 17 to 21)**											
	<17	0.89^d^ (0.82-0.96)		0.92^b^ (0.86-0.99)		0.90^d^ (0.84-0.97)		0.95 (0.89-1.03)		0.95 (0.89-1.03)	
	≥22	1.04 (0.95-1.14)		1.08 (1.00-1.18)		1.05 (0.97-1.14)		1.10^b^ (1.01-1.19)		1.12^c^ (1.03-1.21)	
**Sleep disturbance (reference: <12)**											
	≥12	0.88^d^ (0.80-0.96)		0.94 (0.87-1.02)		0.93 (0.86-1.01)		0.97 (0.89-1.05)		0.98 (0.90-1.07)	
**Survey response time (min; reference: <15)**											
	15 to 30	0.99 (0.92-1.06)		0.97 (0.91-1.03)		0.97 (0.91-1.04)		0.98 (0.92-1.04)		1.02 (0.95-1.09)	
	≥30	0.96 (0.83-1.12)		0.95 (0.83-1.08)		0.98 (0.86-1.11)		1.02 (0.90-1.16)		1.04 (0.91-1.19)	
**Usability of the survey website (reference: neutral)**											
	Satisfied	0.90^b^ (0.81-0.99)		0.83^c^ (0.76-0.90)		0.85^c^ (0.79-0.93)		0.82^c^ (0.75-0.89)		0.85^c^ (0.78-0.93)	
	Unsatisfied	0.84 (0.65-1.10)		0.82 (0.65-1.04)		0.89 (0.71-1.11)		1.02 (0.82-1.28)		0.95 (0.75-1.19)	
**Comprehensive feelings on the survey (reference: neutral)**											
	Satisfied	1.02 (0.92-1.12)		1.04 (0.96-1.14)		1.01 (0.93-1.10)		0.97 (0.89-1.05)		0.92 (0.84-1.00)	
	Unsatisfied	1.07 (0.86-1.34)		1.13 (0.93-1.38)		1.04 (0.86-1.26)		1.214^b^ (1.00-1.47)		1.25^b^ (1.02-1.54)	

^a^Nagelkerke *R*^2^ values: 0.015 (second survey), 0.018 (third survey), 0.018 (fourth survey), 0.018 (fifth survey), 0.019 (sixth survey). Model fit *P* values were all <.001 across surveys.

^b^*P*<.05.

^c^*P*<.001.

^d^*P*<.01.

**Table 3:** Fully adjusted logistic regression of 7th to 11th surveys for nonresponse in the Korea Nurses’ Health Study participants^a^.

Variables		7th survey, OR (95% CI)	8th survey, OR (95% CI)	9th survey, OR (95% CI)	10th survey, OR (95% CI)	11th survey, OR (95% CI)
**Age (y; reference: 20 to 29)**						
	30 to 39	0.84^b^ (0.78-0.90)	0.88^b^ (0.82-0.94)	0.82^b^ (0.76-0.88)	0.93^c^ (0.87-1.00)	0.82^b^ (0.76-0.88)
	≥40	1.02 (0.89-1.16)	1.04 (0.91-1.19)	0.85^b^ (0.74-0.97)	1.02 (0.90-1.17)	0.97 (0.85-1.10)
**Geographical region (reference: Seoul)**						
	Metropolitan cities	1.09^c^ (1.01-1.17)	1.02 (0.95-1.10)	1.05 (0.97-1.13)	0.97 (0.90-1.05)	0.98 (0.91-1.06)
	Others	1.03 (0.96-1.11)	0.98 (0.92-1.06)	1.03 (0.96-1.11)	1.00 (0.93-1.07)	0.99 (0.92-1.06)
**Education (reference: associate degree)**						
	Bachelor degree^b^	0.81 (0.76-0.87)	0.84 (0.79-0.90)	0.85 (0.80-0.91)	0.85 (0.80-0.90)	0.82 (0.77-0.87)
	Master degree or higher	0.80^d^ (0.70-0.91)	0.87^c^ (0.76-0.99)	0.84^c^ (0.74-0.96)	0.79^b^ (0.69-0.90)	0.82^d^ (0.72-0.94)
**Hospital size (reference: ≥300 beds)**						
	<300 beds	1.05 (0.97-1.13)	1.02 (0.94-1.09)	1.07 (1.00-1.15)	0.99 (0.92-1.06)	1.01 (0.94-1.09)
**Job role (reference: charge or staff nurse)**						
	Manager or head nurse	1.11 (0.95-1.31)	1.04 (0.89-1.22)	1.03 (0.88-1.21)	0.96 (0.82-1.13)	1.03 (0.88-1.21)
**Work unit type (reference: general ward)**						
	Special unit	1.06 (0.99-1.13)	1.03 (0.97-1.10)	1.05 (0.99-1.12)	1.04 (0.98-1.11)	1.07^c^ (1.01-1.15)
	Delivery room or others	0.94 (0.87-1.03)	1.00 (0.92-1.09)	0.97 (0.89-1.05)	0.96 (0.88-1.04)	0.98 (0.90-1.06)
**Depressive symptoms (reference: <10)**						
	≥10	0.93 (0.86-1.00)	0.96 (0.89-1.04)	0.98 (0.91-1.06)	0.93 (0.86-1.00)	0.96 (0.89-1.04)
**Stress (reference: <11)**						
	≥11	1.20^c^ (1.02-1.41)	1.20^c^ (1.03-1.40)	1.09 (0.94-1.28)	1.16 (0.99-1.35)	1.10 (0.95-1.28)
**Fatigue (reference: approximately 17 to 21)**						
	<17	0.96 (0.89-1.03)	1.01 (0.94-1.08)	1.00 (0.93-1.07)	1.04 (0.97-1.12)	0.96 (0.90-1.04)
	≥22	1.05 (0.97-1.14)	1.05 (0.97-1.13)	1.03 (0.96-1.12)	1.07 (0.94-1.11)	1.00 (0.92-1.08)
**Sleep disturbance (reference: <12)**						
	≥12	0.96 (0.89-1.05)	0.98 (0.91-1.06)	1.01 (0.93-1.09)	1.03 (0.95-1.12)	1.02 (0.94-1.11)
**Survey response time (min; reference: <15)**						
	15 to 30	1.03 (0.97-1.10)	1.02 (0.95-1.08)	0.99 (0.93-1.06)	0.99 (0.93-1.05)	0.98 (0.92-1.05)
	≥30	0.98 (0.86-1.11)	1.02 (0.90-1.16)	0.90 (0.79-1.02)	0.99 (0.87-1.13)	1.01 (0.88-1.14)
**Usability of the survey website (reference: neutral)**						
	Satisfied	0.83^b^ (0.76-0.90)	0.84^b^ (0.77-0.92)	0.86^b^ (0.79-0.93)	0.89^d^ (0.82-0.97)	0.89^d^ (0.82-0.97)
	Unsatisfied	1.15 (0.91-1.45)	1.16 (0.93-1.46)	1.14 (0.91-1.42)	1.30^c^ (1.04-1.63)	1.06 (0.85-1.33)
**Comprehensive feelings on the survey (reference: neutral)**						
	Satisfied	0.93 (0.86-1.02)	0.96 (0.88-1.04)	0.92 (0.85-1.01)	0.93 (0.86-1.02)	0.93 (0.85-1.01)
	Unsatisfied	1.26^c^ (1.03-1.54)	1.21 (1.00-1.47)	1.17 (0.97-1.42)	1.13 (0.93-1.37)	1.15 (0.95-1.40)

^a^Nagelkerke *R*^2^ values: 0.015 (7th survey), 0.009 (8th survey), 0.012 (9th survey), 0.008 (10th survey), 0.011 (11th survey). Model fit *P* values were all <.001 across surveys.

^b^*P*<.001.

^c^*P*<.05.

^d^*P*<.01.

Geographical region was significantly related to nonresponse until the seventh survey, and hospital size was significant until the fifth survey. In the second survey, nurses working in metropolitan cities (OR 1.16, 95% CI 1.06-1.27) and other areas (OR 1.17, 95% CI 1.08-1.27) were more likely to be nonrespondents than those working in Seoul. This was consistently significant up to the sixth survey in other areas (OR 1.13, 95% CI 1.04-1.21) and up to the seventh survey in metropolitan cities (OR 1.09, 95% CI 1.01-1.17). Nurses working in hospitals with fewer than 300 beds were more likely to be nonrespondents than their counterparts in the second survey (OR 1.40, 95% CI 1.29-1.52), which was consistently significant up to the fifth survey (OR 1.15, 95% CI 1.07-1.24).

Psychological factors, including fatigue, stress, and sleep disturbance, were significant non–response-related factors in some follow-up surveys, but did not show consistency across follow-up surveys.

Finally, it has been reported that the demographic characteristics of participants, such as gender and ethnicity, may influence their responses to cohort studies [17]. However, the Korea Nurses’ Health Study participants comprised only female Korean nurses, leaving little room to explain the influence of gender and ethnicity on nonresponse in community-based cohort studies and future studies.

## Discussion

### Principal Findings

In this study, we reported the nonresponse rate and associated factors for more than 10 follow-up surveys. Our findings are crucial for informing future research on the factors that should be considered when designing and recruiting cohort studies, especially for women and nurses. Cohort study results should be interpreted carefully, considering random nonresponse factors. Thus, researchers must report nonresponses that occur during the study period when synthesizing studies using quality evaluation to provide a high level of evidence [32].

As of March 2025, the ongoing Korea Nurses’ Health Study has conducted more than 10 follow-up surveys, with nonresponse rates ranging from 25% (5258/20,613) to 61% (12,620/20,613) and averaging around 50% (10,306/20,613). Unexpectedly, nonresponse peaked at the sixth survey from September 2017 to September 2019, although there was no change in the recruitment method. This may be attributed to reduced promotional activities following a change in the managing research institution in 2018. After the change in the research institute, we started the seventh survey and focused on promotional activities to increase response rates.

In this study, the age and education level of the nurses and the perceived usability of the survey website from the baseline survey were nonresponse factors throughout the 11th follow-up survey. Fewer nurses in their 20s at the baseline survey responded to the follow-up surveys than those in their 30s. This may be related to career stability and professional confidence, which often increase with experience [33-35]. Conversely, younger nurses may face greater challenges when transitioning from student to professional nurses [36,37], considering that the mean age of newly graduated nurses is 24.7 years in Korea [38], which is much younger than the 29 years in the United States [39]. Notably, nurses aged more than 40 years did not show significantly different nonresponse rates from those in their 20s, indicating that factors other than age, such as career development, leadership roles in the organization, and life-work balance, may influence survey response at follow-up. These findings suggest the need for supportive strategies tailored to different career stages to enhance participation in longitudinal studies.

Consistent with previous studies [25,40-43], educational level was associated with nonresponse. Nurses with associate degrees were less likely to respond to follow-up surveys than those with higher education levels. Targeting more participants with less education may be a strategy for retaining this group in follow-up surveys of cohort studies. Contrary to our findings, 1 longitudinal study that investigated nonresponse to follow-up surveys among an aging population did not report education as a factor related to nonresponse [26]. However, the influence of education may not be applicable to older adults in cohort studies.

In addition, nurses working in hospitals with more than 300 beds and those located in Seoul were more likely to respond to the follow-up surveys through the fifth and sixth rounds, respectively, compared to their counterparts. Most large hospitals are located in Seoul, and university-affiliated hospitals and large corporation-operated hospitals may have more professional and systematic organizational support for nurses [44,45], which might have influenced their value in health research. However, because nurses may change their jobs or relocate, baseline information on location and workplace may not retain its predictive value in later follow-ups.

Although the association between psychological factors, such as depression, stress, fatigue, sleep disturbance, and nonresponse in the follow-up surveys, was not consistent in this study, there is some evidence of psychological factors influencing the follow-up surveys in the literature. Studies have reported that experiencing stressful life events has been shown to result in lower subsequent follow-up responses [40], and respondents reported better subjective health and healthier lifestyle behaviors than nonrespondents [40,46]. Therefore, further systematic research on this topic is required.

Interestingly, the time taken to complete the survey was not a significant factor in nonresponse; rather, the perceived usability of the survey website was related to nonresponse. This suggests that participants were willing to return to the survey regardless of the length of time spent; however, technical issues in completing the surveys might be a barrier to participation in future research. The presentation of survey websites is an important but often overlooked part of data collection, as it can influence not only the response rate but also the measurement error [47]. Researchers who use websites to deliver surveys in cohort studies should carefully consider the wording and display of questions, including question ordering, to increase the response rates [48].

Strategies for participant recruitment and retention in longitudinal research should be specifically developed to minimize the dropout risk. Current suggestions for designing cohort studies focus on recruiting and maintaining retention while considering the education, age, and psychometric characteristics of registered nurses. Further research on ethnic minority groups [49] is required worldwide. The factors associated with the nonresponse rates in this study should also be considered when designing cohort studies involving women. As nonresponse rates differed according to demographic factors such as age, education, and geographical region, tailored strategies should be developed to lower the nonresponse rate of participants with specific demographic characteristics. Examples of these strategies include posting flyers in certain regions, such as small cities, retrieving participants, and advertising the research through social networking services to gain access to those who are younger and who do not live or work in metropolitan areas.

### Limitations

The data were measured only at baseline, which limited the ability to assess the influence of geographical location changes over time, such as participants moving from Seoul to metropolitan or urban areas in subsequent survey waves. Furthermore, the statistical analysis of confounding variables may yield further information regarding nonresponse. We could not identify participants who died during the follow-up period. However, participants who had left the nursing profession were included because this study focused on women’s health rather than their professional roles. In addition, 3 to 3.7% of participants could not be reached due to lost contact information.

### Conclusions

The nonresponse factors identified by the Korea Nurses’ Health Study during the 10-year follow-up survey were age, education, and comprehensive feelings about the survey. We suggest that future research examine the extent of nonresponse bias and its potential impact on the observed associations to further enhance the validity and reliability of the findings. The implementation of weighting methods to account for nonresponse may also be considered in subsequent analyses. Customized strategies that reflect these factors should be developed and tested in future studies to increase follow-up survey response rates.

## Appendix

Multimedia Appendix 1 Differences in baseline characteristics from second to sixth and from 7th to 11th surveys between respondent and nonrespondent groups among Korea Nurses’ Health Study participants (N=20,613).

## Abbreviations

OR: odds ratio
PSS: Perceived Stress Scale
