# Supplementary material for: Factors Affecting Nonresponse Among Female Participants in the Korea Nurses’ Health Study: Longitudinal Cohort Survey Study
Source: JMIR Public Health Surveill. 2025 Oct 20;11:e68038. doi: 10.2196/68038 (PMC12583941; doi:10.2196/68038)
Supplement: Multimedia Appendix 1 [file publichealth_v11i1e68038_app1.docx]

Table S1. Differences in baseline characteristics between response and non-response groups in KNHS^a^ participants (N = 20,613)

| Variables | 2nd survey | | 3rd survey | | 4th survey | | 5th survey | | 6th survey | |
| --- | --- | --- | --- | --- | --- | --- | --- | --- | --- | --- |
|  | Response  (N=15,355) | Non-response  (N=5,258) | Response  (N=12,848) | Non-response  (N=7,765) | Response (N=10,650) | Non-response  (N=9,963) | Response (N=11,527) | Non-response  (N=9,086) | Response  (N=7,993) | Non-response  (N=12,620) |
|  | n (%) | n (%) | n (%) | n (%) | n (%) | n (%) | n (%) | n (%) | n (%) | n (%) |
| Age |  |  |  |  |  |  |  |  |  |  |
| 20-29 | 8941 (58.2) | 3114 (59.2) | 7376 (57.4) | 4679 (60.3) | 6060 (56.9) | 5995 (60.2) | 6505 (56.4) | 5550 (61.1) | 4357 (54.5) | 7698 (61.0) |
| 30-39 | 5088 (33.1) | 1754 (33.4) | 4350 (33.9) | 2492 (32.1) | 3737 (35.1) | 3105 (31.2) | 4045 (35.1) | 2797 (30.8) | 2933 (36.7) | 3909 (31.0) |
| ≥40 | 1326 (8.6) | 390 (7.4) | 1122 (8.7) | 594 (7.6) | 853 (8.0) | 863 (8.7) | 977 (8.5) | 739 (8.1) | 703 (8.8) | 1013 (8.0) |
| *p*-value | .021* | | <.0001*** | | <.0001*** | | <.0001*** | | <.0001*** | |
| Geographical region |  |  |  |  |  |  |  |  |  |  |
| Seoul | 4863 (31.7) | 1450 (27.6) | 4183 (32.5) | 2130 (27.4) | 3573 (33.5) | 2740 (27.5) | 3783 (32.8) | 2530 (27.8) | 2652 (33.2) | 3661 (29.0) |
| Metropolitan cities | 4756 (31.0) | 1718 (32.7) | 3916 (30.5) | 2558 (32.9) | 3106 (29.2) | 3368 (33.8) | 3457 (30.0) | 3017 (33.2) | 2390 (29.9) | 4084 (32.4) |
| Other | 5735 (37.3) | 2090 (39.7) | 4748 (37.0) | 3077 (39.6) | 3970 (37.3) | 3855 (38.7) | 4286 (37.2) | 3539 (39.0) | 2950 (36.9) | 4875 (38.6) |
| *p*-value | <.0001*** | | <.0001*** | | <.0001*** | | <.0001*** | | <.0001*** | |
| Education |  |  |  |  |  |  |  |  |  |  |
| Associate’s degree | 7047 (45.9) | 2724 (51.8) | 5778 (45.0) | 3993 (51.4) | 4728 (44.7) | 5043 (50.6) | 5114 (44.4) | 4657 (51.3) | 3413 (42.7) | 6358 (50.4) |
| Bachelor’s degree | 7099 (46.2) | 2217 (42.2) | 6018 (46.8) | 3298 (42.3) | 5055 (47.5) | 4261 (42.8) | 5468 (47.4) | 3848 (42.4) | 3899 (48.8) | 5417 (42.9) |
| Master’s or higher | 1208 (7.9) | 317 (6.0) | 1051 (8.2) | 474 (6.1) | 867 (8.1) | 658 (6.6) | 945 (8.2) | 580 (6.4) | 681 (8.5) | 844 (6.7) |
| *p*-value | <.0001*** | | <.0001*** | | <.0001*** | | <.0001*** | | <.0001*** | |
| Hospital size |  |  |  |  |  |  |  |  |  |  |
| <300 beds | 3035 (19.8) | 1378 (33.0) | 2481 (19.3) | 1932 (24.9) | 2065 (19.4) | 2348 (23.6) | 2308 (20.0) | 2105 (23.2) | 1651 (20.7) | 2762 (21.9) |
| ≥300 beds | 11643(75.8) | 3632 (69.0) | 9769 (76.0) | 5506 (70.9) | 8040 (75.5) | 7235 (72.6) | 8619 (74.8) | 6656 (73.2) | 5878 (73.5) | 9397 (74.5) |
| *p*-value | <.0001*** | | <.0001*** | | <.0001*** | | <.0001*** | | .199 | |
| Job role |  |  |  |  |  |  |  |  |  |  |
| Manager/head nurse | 734 (4.8) | 245 (4.7) | 625 (4.9) | 354 (4.6) | 480 (4.5) | 499 (5.0) | 555 (4.8) | 424 (4.7) | 387 (4.8) | 592 (4.7) |
| Charge/staff nurse | 14334(93.3) | 4958 (94.3) | 11953(93.0) | 7339 (94.5) | 9929 (93.2) | 9363 (94.0) | 10738(93.1) | 8554 (94.1) | 7432 (93.0) | 11860 (94.0) |
| *p*-value | .651 | | .251 | | .140 | | .531 | | .545 | |
| Work unit type |  |  |  |  |  |  |  |  |  |  |
| Special unit | 4705 (30.6) | 1595 (30.3) | 3961 (30.8) | 2339 (30.1) | 3252 (30.5) | 3048 (30.6) | 3474 (30.1) | 2826 (31.1) | 2383 (29.8) | 3917 (31.0) |
| General ward | 7228 (47.0) | 2435 (46.3) | 6004 (46.7) | 3659 (47.1) | 4955 (46.5) | 4708 (47.3) | 5411 (46.9) | 4252 (46.8) | 1214 (15.2) | 1934 (15.3) |
| Delivery room/others | 2260 (14.7) | 888 (16.9) | 1878 (14.6) | 1270 (16.4) | 1568 (14.7) | 1580 (15.9) | 1724 (15.0) | 1424 (15.7) | 3746 (46.9) | 5917 (46.9) |
| *p*-value | .002** | | .009** | | .237 | | .372 | | .482 | |
| Depressive symptoms |  |  |  |  |  |  |  |  |  |  |
| <10 | 11092(72.2) | 3760 (71.5) | 9313 (72.5) | 5539 (71.3) | 7728 (72.6) | 7124 (71.5) | 8366 (72.6) | 6486 (71.4) | 5828 (72.9) | 9024 (71.5) |
| ≥10 | 4242 (27.6) | 1492 (28.4) | 3519 (27.4) | 2215 (28.5) | 2907 (27.3) | 2827 (28.4) | 3149 (27.3) | 2585 (28.5) | 2156 (27.0) | 3578 (28.4) |
| *p*-value | .301 | | .078 | | .087 | | .069 | | .031* | |
| Stress |  |  |  |  |  |  |  |  |  |  |
| <11 | 14787(96.3) | 5015 (95.4) | 12379(96.3) | 7423 (95.6) | 10260(96.3) | 9542 (95.8) | 11112(96.4) | 8690 (95.6) | 7722 (96.6) | 12080 (95.7) |
| ≥11 | 568 (3.7) | 243 (4.6) | 469 (3.7) | 342 (4.4) | 390 (3.7) | 421 (4.2) | 415 (3.6) | 396 (4.4) | 271 (3.4) | 540 (4.3) |
| *p*-value | .003** | | .008** | | .038* | | .006** | | .001** | |
| Fatigue |  |  |  |  |  |  |  |  |  |  |
| <17 | 6178 (40.2) | 1967 (37.4) | 5216 (40.6) | 2929 (37.7) | 4354 (40.9) | 3791 (38.0) | 4680 (40.6) | 3465 (38.1) | 3300 (41.3) | 4845 (38.4) |
| 17~21 | 4781 (31.1) | 1703 (32.4) | 4017 (31.3) | 2467 (31.7) | 3304 (31.0) | 3180 (31.9) | 3624 (31.4) | 2860 (31.5) | 2515 (31.5) | 3969 (31.5) |
| ≥22 | 4394 (28.6) | 1586 (30.2) | 3613 (28.1) | 2367 (30.5) | 2990 (28.0) | 2990 (23.0) | 3221 (27.9) | 2759 (30.4) | 2177 (27.2) | 3803 (30.1) |
| *p*-value | .001** | | <.0001*** | | <.0001*** | | <.0001*** | | <.0001*** | |
| Sleep disturbance |  |  |  |  |  |  |  |  |  |  |
| <12 | 12330(80.3) | 4275 (81.3) | 10352(80.6) | 6253 (80.5) | 8576 (80.5) | 8029 (80.6) | 9307 (80.7) | 7298 (80.3) | 6487 (81.1) | 10118 (80.2) |
| ≥12 | 3023 (19.7) | 982 (18.7) | 2494 (19.4) | 1511 (19.5) | 2072 (28.8) | 1933 (19.4) | 2218 (19.2) | 1787 (19.7) | 1505 (18.8) | 2500 (19.8) |
| *p*-value | .111 | | .942 | | .930 | | .446 | | .083 | |
| Survey response time |  |  |  |  |  |  |  |  |  |  |
| <15min | 5034 (32.8) | 1767 (33.6) | 4183 (32.6) | 2618 (33.7) | 3467 (32.3) | 3334 (33.5) | 3752 (32.5) | 3049 (33.6) | 2624 (32.8) | 4177 (33.1) |
| 15~30min | 9250 (60.2) | 3154 (60.0) | 7758 (60.4) | 4646 (59.8) | 6438 (60.4) | 5966 (60.0) | 6992 (60.7) | 5412 (59.6) | 4833 (60.5) | 7571 (60.0) |
| ≥30min | 1071 (7.0) | 336 (6.4) | 907 (7.1) | 500 (6.4) | 745 (7.0) | 662 (6.6) | 783 (6.8) | 624 (6.9) | 536 (6.7) | 871 (6.9) |
| *p*-value | .246 | | .085 | | .287 | | .271 | | .754 | |
| Usability of the survey website |  |  |  |  |  |  |  |  |  |  |
| Satisfied | 7886 (51.4) | 2604 (49.5) | 6695 (52.1) | 3795 (48.9) | 5578 (52.4) | 4912 (49.3) | 6151 (53.7) | 4339 (47.8) | 4342 (54.3) | 6148 (48.7) |
| Neutral | 7095 (46.2) | 2544 (48.4) | 5834 (45.4) | 3805 (49.0) | 4803 (45.1) | 4836 (48.5) | 5112 (44.3) | 4527 (50.0) | 3468 (43.4) | 6171 (48.9) |
| Unsatisfied | 374 (2.4) | 109 (2.1) | 319 (2.5) | 164 (2.11) | 269 (2.5) | 214 (2.1) | 264 (2.3) | 219 (2.4) | 183 (2.3) | 300 (2.4) |
| *p*-value | .013* | | <.0001*** | | <.0001*** | | <.0001*** | | <.0001*** | |
| Comprehensive feelings  on the survey |  |  |  |  |  |  |  |  |  |  |
| Satisfied | 6262 (40.8) | 2092 (39.8) | 5294 (41.2) | 3060 (39.4) | 4414 (41.4) | 3940 (39.5) | 4880 (42.3) | 3474 (38.2) | 3465 (43.4) | 4889 (38.7) |
| Neutral | 8626 (56.1) | 3020 (57.4) | 7162 (55.7) | 4484 (57.7) | 5904 (55.4) | 5742 (57.6) | 6325 (54.9) | 5321 (58.6) | 4312 (53.9) | 7334 (58.1) |
| Unsatisfied | 467 (3.0) | 145 (2.8) | 392 (3.1) | 220 (2.8) | 332 (3.1) | 280 (2.8) | 322 (2.8) | 290 (3.2) | 216 (2.7) | 396 (3.1) |
| *p*-value | .210 | | .018* | | .005** | | <.0001*** | | <.0001*** | |

^a^KNHS, Korea nurses’ health study

^*^*p*<.05, ^**^*p*<.01, ^***^*p*<.001.

Table S1. Differences in baseline characteristics between response and non-response groups in KNHS^a^ participants (Continued)

| Variables | 7th survey | | 8th survey | | 9th survey | | 10th survey | | 11th survey | |
| --- | --- | --- | --- | --- | --- | --- | --- | --- | --- | --- |
|  | Response (N=8,658) | Non-response  (N=11,955) | Response  (N=10,253) | Non-response  (N=10,360) | Response (N=10,656) | Non-response  (N=9,957) | Response (N=10,110) | Non-response  (N=10,503) | Response (N=11,256) | Non-response  (N=10,357) |
|  | n (%) | n (%) | n (%) | n (%) | n (%) | n (%) | n (%) | n (%) | n (%) | n (%) |
| Age |  |  |  |  |  |  |  |  |  |  |
| 20-29 | 4882 (56.4) | 7173 (60.0) | 5872 (57.3) | 6183 (59.7) | 5954 (55.9) | 6101 (61.3) | 5795 (57.3) | 6260 (59.6) | 5746 (51.0) | 6309 (60.9) |
| 30-39 | 3077 (35.5) | 3765 (31.5) | 3557 (34.7) | 3285 (31.7) | 3755 (35.2) | 3087 (31.0) | 3457 (34.2) | 3385 (32.2) | 3636 (32.3) | 3206 (31.0) |
| ≥40 | 699 (8.1) | 1017 (8.5) | 824 (8.0) | 892 (8.6) | 947 (8.9) | 769 (7.7) | 858 (8.5) | 858 (8.2) | 874 (7.8) | 842 (8.1) |
| *p*-value | <.0001*** | | <.0001*** | | <.0001*** | | .004** | | <.0001*** | |
| Geographical region |  |  |  |  |  |  |  |  |  |  |
| Seoul | 2741 (31.7) | 3572 (29.9) | 3160 (30.8) | 3153 (30.4) | 3325 (31.2) | 2988 (30.0) | 3107 (30.7) | 3206 (30.5) | 3147 (28.0) | 3166 (30.6) |
| Metropolitan cities | 2658 (30.7) | 3816 (31.9) | 3199 (31.2) | 3275 (31.6) | 3309 (31.1) | 3165 (31.8) | 3187 (31.5) | 3287 (31.3) | 3228 (28.7) | 3246 (31.3) |
| Other | 3258 (38.0) | 4567 (38.2) | 3893 (38.0) | 3932 (38.0) | 4021 (37.8) | 3804 (38.2) | 3815 (37.7) | 4010 (38.2) | 3880 (34.5) | 3945 (38.1) |
| *p*-value | .018* | | .768 | | .167 | | .808 | | .931 | |
| Education |  |  |  |  |  |  |  |  |  |  |
| Associate’s degree | 3821 (44.1) | 5950 (49.8) | 4645 (45.3) | 5126 (49.5) | 4786 (44.9) | 4985 (49.8) | 4549 (45.0) | 5222 (49.7) | 4574 (40.6) | 5197 (50.2) |
| Bachelor’s degree | 4147 (47.9) | 5169 (43.2) | 4824 (47.0) | 4492 (43.4) | 4997 (46.9) | 4319 (43.4) | 4754 (47.0) | 4562 (43.4) | 4866 (43.2) | 4450 (43.0) |
| Master’s or higher | 689 (8.0) | 836 (7.0) | 783 (7.6) | 742 (7.2) | 872 (8.2) | 653 (6.6) | 806 (8.0) | 719 (6.8) | 815 (7.2) | 710 (6.9) |
| *p*-value | <.0001*** | | <.0001*** | | <.0001*** | | <.0001*** | | <.0001*** | |
| Hospital size |  |  |  |  |  |  |  |  |  |  |
| <300 beds | 1818 (21.0) | 2595 (21.7) | 2172 (21.2) | 2241 (21.6) | 2220 (20.8) | 2193 (22.0) | 2147 (21.2) | 2266 (21.6) | 2194 (19.5) | 2219 (21.4) |
| ≥300 beds | 6418 (74.1) | 8857 (74.1) | 7603 (74.2) | 7672 (74.1) | 7866 (74.8) | 7409 (74.4) | 7431 (73.5) | 7844 (74.7) | 7517 (66.8) | 7758 (74.9) |
| *p*-value | .332 | | .516 | | .166 | | .997 | | .554 | |
| Job role |  |  |  |  |  |  |  |  |  |  |
| Manager/head nurse | 391 (4.5) | 588 (4.9) | 472 (4.6) | 507 (4.9) | 529 (5.0) | 450 (4.5) | 496 (4.9) | 483 (4.6) | 507 (4.5) | 472 (4.6) |
| Charge/staff nurse | 8106 (93.6) | 11186 (93.6) | 9606 (93.7) | 9686 (93.5) | 9946 (93.3) | 9346 (93.9) | 9442 (93.4) | 9850 (93.8) | 9582 (85.1) | 9710 (93.8) |
| *p*-value | .207 | | .342 | | .132 | | .293 | | .196 | |
| Work unit type |  |  |  |  |  |  |  |  |  |  |
| Special unit | 2557 (29.5) | 3743 (31.3) | 3096 (30.2) | 3204 (31.0) | 3183 (29.9) | 3117 (31.3) | 3027 (30.0) | 3273 (31.2) | 3048 (27.1) | 3252 (31.4) |
| General ward | 4050 (46.8) | 5613 (47.0) | 1586 (15.5) | 1562 (15.1) | 4997 (46.9) | 4666 (46.9) | 1594 (15.8) | 1554 (14.8) | 1620 (14.4) | 1528 (14.8) |
| Delivery room/others | 1387 (16.0) | 1761 (14.7) | 4820 (47.0) | 4843 (46.7) | 1682 (15.8) | 1466 (14.7) | 4736 (46.8) | 4927 (46.9) | 4816 (42.8) | 4847 (46.8) |
| *p*-value | .005** | | .475 | | .027* | | .059 | | .016* | |
| Depressive symptoms |  |  |  |  |  |  |  |  |  |  |
| <10 | 6269 (72.4) | 8583 (71.8) | 7417 (72.3) | 7435 (71.8) | 7750 (72.7) | 7102 (71.3) | 7285 (72.1) | 7567 (72.0) | 7446 (66.2) | 7406 (71.5) |
| ≥10 | 2379 (27.5) | 3355 (28.0) | 2823 (27.5) | 2911 (28.1) | 2896 (27.1) | 2838 (28.5) | 2810 (27.8) | 2924 (27.8) | 2800 (24.9) | 2934 (28.3) |
| *p*-value | .353 | | .367 | | .032* | | .954 | | .094 | |
| Stress |  |  |  |  |  |  |  |  |  |  |
| <11 | 8360 (96.6) | 11442 (95.7) | 9889 (96.4) | 9913 (95.7) | 10269 (96.4) | 9533 (95.7) | 9742 (96.4) | 10060 (95.8) | 9883 (87.8) | 9919 (95.8) |
| ≥11 | 298 (3.4) | 513 (4.3) | 364 (3.6) | 447 (4.3) | 387 (3.6) | 424 (4.3) | 368 (3.6) | 443 (4.2) | 373 (3.3) | 438 (4.2) |
| *p*-value | .002** | | .005** | | .022* | | .033* | | .029* | |
| Fatigue |  |  |  |  |  |  |  |  |  |  |
| <17 | 3520 (40.7) | 4625 (38.7) | 4082 (39.8) | 4063 (39.2) | 4273 (40.1) | 3872 (38.9) | 4051 (40.1) | 4094 (39.0) | 4129 (36.7) | 4016 (38.8) |
| 17~21 | 2713 (31.3) | 3771 (31.5) | 3259 (31.8) | 3225 (31.1) | 3359 (31.5) | 3125 (31.4) | 3145 (31.1) | 3339 (31.8) | 2930 (26.0) | 3050 (29.4) |
| ≥22 | 2424 (28.0) | 3556 (29.7) | 2911 (28.4) | 3069 (29.6) | 3023 (28.4) | 2957 (29.7) | 2912 (28.8) | 3068 (29.2) | 3195 (28.4) | 3289 (31.8) |
| *p*-value | .006** | | .145 | | .079 | | .271 | | .089 | |
| Sleep disturbance |  |  |  |  |  |  |  |  |  |  |
| <12 | 6987 (80.7) | 9618 (80.5) | 8271 (80.7) | 8334 (80.4) | 8636 (81.0) | 7969 (80.0) | 8177 (80.9) | 8428 (80.2) | 8319 (73.9) | 8286 (80.0) |
| ≥12 | 1670 (19.3) | 2335 (19.5) | 1981 (19.3) | 2024 (19.5) | 2019 (18.9) | 1986 (19.9) | 1931 (19.1) | 2074 (19.7) | 1935 (17.2) | 2070 (20.0) |
| *p*-value | .668 | | .699 | | .070 | | .242 | | .043* | |
| Survey response time |  |  |  |  |  |  |  |  |  |  |
| <15min | 2887 (33.3) | 3914 (32.7) | 3397 (33.1) | 3404 (32.9) | 3475 (32.6) | 3326 (33.4) | 3325 (32.9) | 3476 (33.1) | 3363 (29.9) | 3438 (33.2) |
| 15~30min | 5178 (59.8) | 7226 (60.4) | 6166 (60.1) | 6238 (60.2) | 6427 (60.3) | 5977 (60.0) | 6109 (60.4) | 6295 (59.9) | 6192 (54.5) | 6212 (60.0) |
| ≥30min | 593 (6.8) | 814 (6.8) | 690 (6.7) | 717 (6.9) | 754 (7.1) | 653 (6.6) | 676 (6.7) | 731 (7.0) | 701 (6.2) | 706 (6.8) |
| *p*-value | .635 | | .819 | | .215 | | .658 | | .822 | |
| Usability of the survey website |  |  |  |  |  |  |  |  |  |  |
| Satisfied | 4714 (54.4) | 5776 (48.3) | 5487 (53.5) | 5003 (48.3) | 5700 (53.5) | 4790 (48.1) | 5384 (53.3) | 5106 (48.6) | 5462 (48.5) | 5028 (48.5) |
| Neutral | 3773 (43.6) | 5866 (49.0) | 4559 (44.5) | 5080 (49.0) | 4728 (44.4) | 4911 (49.3) | 200 (2.0) | 283 (2.7) | 222 (2.0) | 261 (2.5) |
| Unsatisfied | 171 (2.0) | 312 (2.6) | 207 (2.0) | 276 (2.7) | 228 (2.1) | 255 (2.6) | 4526 (44.8) | 5113 (48.7) | 4572 (40.6) | 5067 (48.9) |
| *p*-value | <.0001*** | | <.0001*** | | <.0001*** | | <.0001*** | | <.0001*** | |
| Comprehensive feelings  on the survey |  |  |  |  |  |  |  |  |  |  |
| Satisfied | 3764 (43.5) | 4590 (38.4) | 4374 (42.7) | 3980 (38.4) | 4556 (42.8) | 3798 (38.1) | 4302 (42.6) | 4052 (38.6) | 4364 (38.8) | 3990 (38.5) |
| Neutral | 4677 (54.0) | 6969 (58.3) | 5611 (54.7) | 6035 (58.3) | 5815 (54.6) | 5831 (58.6) | 267 (2.6) | 345 (3.3) | 279 (2.8) | 333 (3.2) |
| Unsatisfied | 217 (2.5) | 395 (3.3) | 268 (2.6) | 344 (3.3) | 285 (2.7) | 327 (3.3) | 5541 (54.8) | 6105 (58.1) | 5613 (49.9) | 6033 (58.3) |
| *p*-value | <.0001*** | | <.0001*** | | <.0001*** | | <.0001*** | | .0001*** | |

*p<.05, **p<.01, ***p<.001.
